# Homozygous Autosomal Recessive *DIAPH1* Mutation Associated with Central Nervous System Involvement and Aspergillosis: A Rare Case

**DOI:** 10.1155/2022/4142214

**Published:** 2022-09-29

**Authors:** Hossein Esmaeilzadeh, Rafat Noeiaghdam, Leila Johari, Seyed Ali Hosseini, Sayyed Hesamedin Nabavizadeh, Soheila Sadat Alyasin

**Affiliations:** ^1^Allergy Research Center, Department of Pediatrics, School of Medicine, Shiraz University of Medical Sciences, Shiraz, Iran; ^2^School of Medicine, Shiraz University of Medical Sciences, Shiraz, Iran

## Abstract

The *DIAPH1* gene fulfills critical immune and neurodevelopmental roles. It encodes the mammalian Diaphanous-related formin (mDia1) protein, which acts downstream of Rho GTPases to promote F-actin polymerization and stabilize microtubules. During mitosis, this protein is expressed in human neuronal precursor cells and considerably affects spindle formation and cell division. In humans, dominant gain-of-function *DIAPH1* variants cause sensorineural deafness and macrothrombocytopenia (DFNA1), while homozygous *DIAPH1* loss leads to seizures, cortical blindness, and microcephaly syndrome (SCBMS). To date, only 16 patients with SCBMS have been reported, none of whom were from Iran. Furthermore, aspergillosis is yet to be reported in patients with homozygous *DIAPH1* loss, and the link between SCBMS and immunodeficiency remains elusive. In this study, we shed further light on this matter by reporting the clinical, genetic, and phenotypic characteristics of an Iranian boy with a long history of recurrent infections, diagnosed with SCBMS and immunodeficiency (NM_005219.5 c.3145C > *T*; p.R1049X variant) following aspergillosis and SARS-CoV-2 coinfection.

## 1. Introduction

The *DIAPH1* gene fulfills critical immune and neurodevelopmental roles; it encodes the mammalian Diaphanous-related formin (mDia1) protein, which acts downstream of Rho GTPases to promote F-actin polymerization and stabilize microtubules [1–3]. Besides its role in malignancies, the *DIAPH1* gene has been implicated in various Mendelian diseases [4, 5]. Dominant gain-of-function *DIAPH1* variants cause sensorineural deafness and macrothrombocytopenia (DFNA1) [6–8], while homozygous DIAPH1 loss leads to seizures, cortical blindness, and microcephaly syndrome (SCBMS). In this syndrome, seizures usually develop during early infancy and are challenging to manage. Though body measurements may be normal at birth, microcephaly, cortical blindness, and intellectual disability gradually develop, sometimes with stunted growth [9–11].

To date, only 16 patients with SCBMS have been reported, including the following variants: NM_005219: c.2332C > *T*; p. Q778X, c.2769delT; p.F923fs, c.3145C > *T*; p.R1049X; and c.68411G > *A* [9–11]. Only one of the previous studies highlights a clear link between SCBMS and immunodeficiency [11], though some cases with bronchiectasis or recurrent respiratory infections have also been described [9, 10]. Furthermore, aspergillosis isyet to be reported in patients with homozygous *DIAPH1* loss. Herein, we shed further light on this matter by reporting the case of an Iranian boy with a long history of recurrent infections, diagnosed with SCBMS and immunodeficiency (NM_005219.5 c.3145C > *T*; p.R1049X variant) following aspergillosis and SARS-CoV-2 coinfection.

## 2. Case Presentation

In February 2022, we admitted a four-year-old boy, who was a known case of CD4 deficiency, seizure, cortical blindness, and microcephaly, due to fever. On physical examination, the vital signs were stable, with the exception of a 38.7°C temperature. The skin featured a scar from a past vasculitis lesion, and both eyes had no response to light. Upon auscultation of the lungs, bilateral rales were detected. Upon admission, his weight, height and head circumference were 12 kg (z-score: −3.37), 59 cm (z-score: −10.32), and 44 cm, respectively.

The patient had a history of seizures, blindness, and recurrent infections since birth. His birth weight was 2.98 kg, his height was 49 cm, and his head circumference was 34 cm, and the parents were distantly related. Neonatal screening for inborn errors of metabolism and endocrinopathies was normal. His seizures had commenced during early infancy in generalized tonic-clonic form; the patient was on antiseizure medications for a period of time, after which the seizures no longer recurred and the medications were tapered and discontinued. Before admission, the patient was receiving prophylactic cotrimoxazole and fluconazole. He had no family history of immunodeficiency or death of unknown cause. He had previously been hospitalized many times, and a summary of the previous five admissions is presented in Table 1. In terms of surgical history, the patient underwent a right posterior lateral thoracotomy and right upper lobectomy when he was one and a half years old due to congenital lobar emphysema. Due to the known immunodeficiency status, broad-spectrum antibiotics (intravenous vancomycin and meropenem) were initiated upon admission.

In the laboratory workup, the following findings were significant: WBC 13,700/*μ*l (62% lymphocytes), Hb 11.1 g/dl, Plt 475,000/*μ*l, ESR 65 mm/hr, CRP 38 mg/l, D-dimer 4,360 ng/mL (high), ferritin 506.4 ng/ml (high), procalcitonin 0.69 ng/ml (equivocal), SARS-CoV-2 IgG 0.2 (negative), SARS-CoV-2 IgM <0.01 (negative), negative serum aspergillus galactomannan Ag, and normal total IgE and HIV Ab/*p*24-Ag. A chest HRCT revealed that the lungs had bilateral diffuse infiltrations in favor of aspergillosis and COVID-19. During the hospital course, the patient developed tachypnea and decreased O2 saturation and was transferred to an intensive care unit following a positive SARS-CoV-2 PCR, where he received remdesivir and dexamethasone. Subsequently, whole blood nested PCR returned positive for aspergillus (and negative for Candida infection), so intravenous voriconazole was prescribed for two weeks. Given that such patients are prone to hematological malignancies, bone marrow flow cytometry phenotyping was requested, which ruled out acute leukemia (less than 3% immature CD34+/CD117+ myeloid cells). Whole exome sequencing (Illumina NovaSeq6000 platform; 100 bp reading length; paired-end sequencing) of peripheral blood DNA was requested, revealing a homozygous c.3145C > *T* variant in the *DIAPH1* gene, reportedly associated with seizures, cortical blindness, and microcephaly (Table 2). Incidentally, the sample was also heterozygous for the c.1129C > *T* variant in the *DNAJC3* gene (Table 2). The patient responded well to treatment and was discharged after two weeks.

## 3. Discussion

Located on chromosome 5 in humans, the *DIAPH1* gene fulfills a crucial role in brain development [10]. This gene is responsible for encoding the RhoA GTPase mDia1 [12, 13], which binds to F-actin to stimulate the addition of actin monomers following activation by RhoA [14]. During mitosis, this protein is expressed in human neuronal precursor cells and considerably affects spindle formation and cell division [10]. Pathogenic variants of the *DIAPH1* gene are involved in a number of diseases: heterozygous gain-of-function variants cause deafness and thrombocytopenia (OMIM 124900) [6, 7] and have also been associated with sporadic moyamoya disease [15]. In contrast, homozygous loss-of-function variants result in SCBMS (OMIM 616632) [9–11].

In his study, we present the characteristics of an Iranian boy with SCBMS and CD4 deficiency, reporting the first case of aspergillosis associated with the NM_005219.5 c.3145C > *T*; p.R1049X variant of homozygous *DIAPH1* loss. The first reported instance of such a mutation affected five siblings in a Saudi Arabian family, who had developmental delay, intellectual disability, blindness, microcephaly, seizures, and stunted growth caused by the c.2332C4T (p.Gln778^†^, RefSeq NM_005219.4) variant. In that report, immunodeficiency was not mentioned, though one patient succumbed to a chest infection at the age of 18 [10]. In the second report on this issue, the variants were detected in four individuals with SCBMS from two unrelated consanguineous families. The first was a boy from the United Arab Emirates who had SCBMS as well as recurrent sinopulmonary infections (causing chronic cough and secretions), though sweat testing and immunological function screenings were normal. He also suffered from wheezing early on and needed supplemental oxygen for seven months. Notably, he had the same variant (c.3145C > *T*; p.R1049X) as our patient. The other three were Omani siblings (two females; one male) who had the c.2769delT; p.F923fs variant of homozygous *DIAPH1* loss. Again, immunodeficiency was not detected, though one female had severe bronchiectasis and died of pneumonia at age 13 [9].

In the only report to have linked homozygous *DIAPH1* loss with SCBMS and immunodeficiency, Kaustio et al. described five Finnish patients homozygous for the NM_005219:c.68411 G > *A* splice-variant and two Omani patients with the NM_005219:c.2769delT; p.F923fs frameshift-variant. In addition to SCBMS, these patients were susceptible to infections due to defective lymphocyte maturation, with three of them developing B-cell lymphoma. Immunophenotyping revealed poor lymphocyte activation and proliferation, defective B-cell maturation, and a lack of naive T cells. The recurrent infections in the patients included otitis media, respiratory infections, candida, mycobacteria, VZV, HSV, EBV, CMV, RSV, MRSA, molluscum contagiosum, recurrent watery diarrhea, rubella vaccine strain skin infection, JCV encephalitis, *Staphylococcus haemolyticus, Elizabethkingia meningoseptica*, and *Streptococcus pneumonia* [11]. Similarly, our patient also had recurrent pulmonary infections and a CMV urinary tract infection, though he also developed COVID-19 and aspergillosis, which were yet to be reported in patients with homozygous *DIAPH1* loss.

Aspergillosis is an opportunistic fungal infection that can be life-threatening in those who are immunocompromised. Factors that render an individual susceptible include neutropenia, corticosteroid use, hematologic malignancies, diabetes, underlying lung disease, and AIDS [16–19], with low CD4 counts being associated with a higher incidence of this condition [20]. In our patient, hematologic malignancies were ruled out and corticosteroids were not routinely used, though underlying lung disease was present in addition to a low CD4 count (920/*μ*l).

According to the Kaustio et al. study, patients with SCBMS can have combined immune deficiency ascytoskeletal organization disorganization and mitochondrial dysfunction are implicated in the pathogenesis of the syndrome [11]. In that study, T cells derived from the patients had impaired adhesion and inefficient microtubule-organizing center translocation to the immune synapse, which is essential for T-cell function. Such a defect has previously been shown in animal models, so immunodeficiency can be expected in such patients given the functions of *DIAPH1* in actin nucleation and microtubule organization and considering that mutations in several cytoskeleton-regulating genes cause primary immunodeficiency [11, 21–23].

An incidental finding reported in our genetic workup was the heterozygous c.1129 C > *T* variant in the *DNAJC3* gene. While homozygous mutations in *DNAJC3* on chromosome 13q32 cause combined cerebellar and peripheral ataxia with hearing loss and diabetes mellitus (ACPHD) [24], there is no evidence to suggest a link between the heterozygous variant found incidentally in our patient and his specific phenotype. Rather, the phenotypic features seen in our case are generally well-aligned with the prior reports on homozygous *DIAPH1* loss, indicating that this *DIAPH1* variant was responsible for the observed phenotype.

The strengths of this study include the characterization of the phenotype related to a very rare form of immunodeficiency for the first time in an Iranian patient. The main limitation is the cross-sectional nature of this report, though the patient remains under our follow-up and longitudinal data can be collected in the future. Our results add evidence in support of the link between homozygous *DIAPH1* loss and T-cell deficiency. Hence, physicians who manage SCBMS patients must be prepared to prevent or treat severe or recurrent infections, and corticosteroid use may need to be limited in these patients to prevent aspergillosis. Further investigations should be conducted into the exact mechanism behind the defective T-cell responses in patients with homozygous *DIAPH1* loss.

## Figures and Tables

**Table 1:** Summary of the previous five hospital admissions of the patient.

Admission	First	Second	Third	Fourth	Fifth
Age (years and months)	1 y 4 m	1 y 6 m	2 y 5 m	2 y 7 m	3 y 7 m

Chief complaint	Productive cough, vomiting	Cough	Fever, cough	Fever	Fever

Diagnosis	Pneumonia	Congenital lobar emphysema	Sepsis vs. pneumonia, CD4 deficiency	COVID-19, sepsis	Suspected sepsis

Duration of admission (days)	7	5	10	14	5

Significant laboratory data	(i) WBC 11,100/*μ*l (ii) Hb 11.6 g/dl (iii) Plt 285,000/*μ*l (iv) Brain MRI: Thin corpus calusom splenium suggestive of mid-central cortical atrophy (v) Spiral chest CT: Multiple cystic structures measuring about 60x55 × 40 mm in mid-zone of right lung contains multiple air-containing cysts with variable size and septation suggestive of type 1 Congenital pulmonary airway malformation. Diffuse patchy air space opacities in both upper and lower lobes with multiple air space nodules suggestive of bronchopneumonia	(i) WBC 7300/*μ*l (ii) Hb 10.4 g/dl (iii) Plt 242,000/*μ*l (iv) Fibrinogen 611 mg/dl (v) ESR 72 mm/hr (vi) CRP >150 mg/l (vii) LDH 801 IU/l (viii) Sweat NaCl test normal (ix) Lung biopsy: emphysematous lung admix by areas of bronchiectasis	(i) WBC 3000/*μ*l (32% lymph.) (ii) Hb 6.6 g/dl (iii) Plt 488,000/*μ*l (iv) Flow cytometry: CD3 71%, CD4 12%^§^, CD8 55%^†^, CD16 23%, CD19 6%^§^, CD56 23%, CD14 16%^†^, CD4/CD8 0.22^§^ (v) Karyotype study on bone marrow culture: normal (46,XY) (vi) Bone marrow aspiration biopsy/immunohistochemistry: erythroid hypoplasia with parvovirus B19 infection; 4–5% immature myeloid cells; about 30% hematogones (vii) Left leg lesion skin biopsy: lymphocytic vasculitis (viii) ANA 3.9 U/ml† (ix) dsDNA (IgG) 26.0 IU/ml^‡^ (x) dsDNA (IgM) 32.7 IU/ml^†^ (xi) ACLA IgM 51.1 U/ml^†^ (xii) Anti Ro (SSA) IU/ml 83.2^†^ (xiii) IgG 16.34 g/l^†^ (xiv) IgM 1.97 g/l^†^ (xv) IgA 1.21 mg/dl^§^ (xvi) IgE 15.8 IU/ml (normal) (xvii) ASMA neg. (xviii) P-ANCA (Anti MPO) neg. (xix) C-ANCA (Anti PR3) 132.0 U/ml^†^	(i) WBC 22,500/*μ*l (ii) Hb 11.3 g/dl (iii) Plt 363,000/*μ*l (iv) ESR: 92 mm/hr (v) CRP>150 mg/l (vi) Procalcitonin 10 ng/ml (vii) Ferritin 225.7 ng/ml (viii) Chest HRCT: COVID-19	(i) WBC 2800/*μ*l (33% lymph.), (ii) Hb 9.3 g/dl (iii) Plt 422,000/*μ*l (iv) D-dimer 570^†^ (v) Procalcitonin 0.82 ng/ml

^†^ High/positive;^‡^ Borderline/equivocal;^§^ Low.

**Table 2:** Details of the results of whole genome sequencing.

Gene and transcript	Variant	Chromosomal location	Associated diseases	OMIM entry no.	Zygosity	CADD score^†^	dbSNP rsID^‡^	Acmg classification	Inheritance
*DIAPH1* NM_005219.5	Exon23 c.3145C > *T* p.R1049X	chr5-140908023G > *A*	Seizures, cortical blindness, and microcephaly	616632	Hom	37	rs863225243	Pathogenic	AR

*Secondary findings*									
*DNAJC3* NM_006260.5	Exon10 c.1129C > *T* p.R377X	chr13-96438246 *C* > *T*	Combined cerebellar and peripheral ataxia, hearing loss, and diabetes	616192	Het	40	rs1403662008	Likely pathogenic	AR

AMCG, American College of Medical Genetics; AR, Autosomal Recessive; Het, Heterozygous; Hom, Homozygous; OMIM, Online Mendelian ^Inheritance^ in Man.^†^ CADD score of 20 means that a variant is among the top 1% of deleterious variants in the human genome. A CADD score of 30 means that the variant is in the top 0.1% and so forth.^‡^ All variants with dbSNP rsID numbers have minor allele frequencies less than 0.5% unless otherwise stated.
